# A Universal Model for Predicting Dynamics of the Epidemics Caused by Special Pathogens

**DOI:** 10.1155/2013/467078

**Published:** 2013-07-24

**Authors:** Alexander G. Bachinsky, Lily Ph. Nizolenko

**Affiliations:** State Research Center of Virology and Biotechnology Vector, Russian Federation, Koltsovo, Novosibirsk 630559, Russia

## Abstract

A universal model intended primarily for predicting dynamics of the mass epidemics (outbreaks) caused by special pathogens is being developed at the State Research Center of Virology and Biotechnology Vector. The model includes the range of major countermeasures: preventive and emergency mass vaccination, vaccination of risk groups as well as search for and isolation/observation of infected cases, contacts, and suspects, and quarantine. The intensity of interventions depends on the availability of the relevant resources. The effect of resource limitations on the development of a putative epidemic of Ebola hemorrhagic fever is demonstrated. The modeling results allow for estimation of the material and human resources necessary for eradication of an epidemic.

## 1. Introduction

Bioterrorism threats as well as emergence of new pandemic and drug-resistant variants of known infections require development of the tools that would adequately predict development of epidemics, assess efficiency of countermeasures, and optimize the efforts directed towards provision of biological safety. Correspondingly, it is necessary to elaborate mathematical models describing dynamics of the socially significant epidemics as well as epidemics caused by the most important special pathogens and the effects of antiepidemic efforts under conditions of limited resources. Use of such models makes it possible to assess the efficiency not only of particular types of antiepidemic activities (AEA) but also of their joint effect based on the available resources and possibility of their mobilization from other localities as well as to estimate the level of biological safety towards the infectious agents involved in simulation.

The relevant literature contains numerous papers describing the mathematical models for studying particular epidemics, outbreaks, and even optimization of interventions, for example, [1–5]. However, we are interested, first and foremost, in creation of a universally accessible product that solves the problems of counteracting epidemics and estimating the level of biological safety of territories (regions) in an integrated manner.

## 2. Materials and Methods

State Research Center of Virology and Biotechnology Vector (SRC VB Vector) is involved in construction of a universal model for epidemics adapted to the special pathogens causing infections, such as smallpox, anthrax, plague, and Ebola, Marburg, Lassa, and Crimean—Congo hemorrhagic fevers. It is assumed that this model is able to simulate the development of any local epidemic of an acute infectious disease with the infection coming from a certain external source or person-to-person contacts independently of the sex, age, and other sociodemographic characteristics as the major transmission pathways. It is also assumed that a relatively simple model structure is sufficient for description of the major specific features in the epidemic dynamics taking into account the fact that the data necessary for adaptation of more intricate structured models are as a rule absent.

The main statements of the model are based on the concept for simulating epidemics proposed by Boev and Makarov [6, 7]. In turn, this concept had originated from the papers by Baroyan and Rvachev, for example, [8]. The model is available at http://vector-epimod.ru/. The most important postulate of this concept consists in that a person successively undergoes several stages of disease development, such as hidden (latent or incubation) period, prodromal stage, and infectious stage. Depending on the time which a person stays in each stage, the functions are specified that determine the probability of a daily transition to the next stage, recovery, lethal outcome, intensity of release of infectious agent (contagiosity), and so on. This concept can be realized as deterministic as well as stochastic models.

The elaborated deterministic model belongs to the SEIRF class similar to [9], where S is designation for susceptible persons, E persons infected in incubation period, I persons infected persons, R persons recovered and, F persons dead. That is, **38** disjoint classes (without taking into account distribution of residence time in each stage) are distinguished within the population of a certain locality. Here are some of these cohorts:

- uninfected persons susceptible to infection;
- uninfected contact persons. Some of them are detected and observed or isolated;
- uninfected persons during establishment of immunity after vaccination;
- the persons displaying primary nonspecific disease symptoms being actually uninfected (suspected, observed, or isolated);
- infected persons in a latent stage;
- infected persons in a latent stage which are detected as contacts (observed or isolated);
- infected persons in a latent stage displaying primary nonspecific disease symptoms;
- patients in a latent stage during establishment of immunity after vaccination;
- infected persons in the second stage of disease (usually, prodromal stage), not detected;
- infected persons in the second stage of disease (usually, prodromal stage) which are observed or isolated;
- infected persons in the final stage of disease, not detected, displaying a mild or a severe form of disease;
- infected persons in the final stage of disease, which are observed or isolated, displaying a mild or a severe form of disease;
- the persons that have recovered and displayed a long-lived immunity, at least for the period of simulated outbreak, or returned to the cohort of immune susceptible persons; and
- those who died of disease.

The characteristics of the representatives of each cohort except for the last two depend of the immune status. 

The computed dynamics of an epidemic depends on the following modeled processes:

- formation of “infection stress” (intensity of infection) because of a potential external infection source and/or infectivity of not isolated infected cases at the second and/or third stages of disease;
- infection of susceptible persons depending on the intensity of infection and immune statuses;
- formation of the cohort of contacts proportional to the number of newly infected sensitive persons;
- infection of contacts with a rate exceeding the rates for other persons;
- transition of infected persons from stage to stage depending on the time they stay in a certain stage;
- recovery, that is, a probabilistic process accelerating with the course of the third stage; typically, the recovered persons are “discarded” from epidemic; however, the persons recovered from some infections are returned back supplementing the cohort of immune persons; and
- death of infected persons, mainly in the third stage; the mortality rate decreases if the detected infected persons are subject to treatment, are immune, or have a mild form of disease.

Three levels of antiepidemic activities (AEA) are taken into account in modeling. These levels mainly determine the rate of detection and isolation (observation) of the infected persons, contacts, and suspects, which, as a rule, are imposed in a successive manner:

1. mild AEA1 implies isolation of the patients that visited a physician. This regime is used when there are unconfirmed suspicions about a possible outbreak onset; that is, when there are confirmed cases in other localities (regions), cases with evident disease signs have been recorded, and contact transmission routes have been detected with a characteristic incubation period; however, a laboratory confirmation is still absent. Contacts and suspects are not purposefully searched for;
2. moderate AEA2 implies quicker isolation of infected cases as well as detection and isolation (observation) of contacts and suspects. This regime is used in the case of confirmed suspicions on the onset of an outbreak, but certain time period is required until all AEA are deployed; and
3. strict AEA3 implies even quicker isolation of infected cases as well as an active search for infected and contacts, including systematic door-to-door visits.

In addition to these three regimes, mass vaccination is specified in the model for some diseases. Mass vaccination is characterized by the width of its coverage and the period when it is implemented. Vaccination of the risk groups including contacts and suspects as well as quarantine of different degrees that decreases the rate of infection can also be used.

All these countermeasures are implemented provided that the corresponding resources are available. The resources include qualified medical/paramedical staff; facilities for isolation/observation of patients, contacts, and suspects; and the stock of prevention tools and drugs. In the case of a deficiency in the corresponding resources, the intensity of the interventions (detection, isolation, vaccination, treatment, etc.) can be weakened down to complete cessation.

Thus, the state of the system at any given moment is specified by the values of all the variables, marked by dot points above.

The transition from state to state with an interval of one day of an epidemic (or disease) is defined as the intensity of the processes listed above too. Thus for each variable, a certain number of representatives of that class go to another class, for example, noninfected ⇒ infected (in proportion to the infectious intensity and proportion of individuals susceptible to infection in the population), “home sick” ⇒ isolated (in proportion to the isolation speed specified for patients having this form of the disease if resources are available), nonimmune persons ⇒ persons, which are in the process of formation of immunity (in proportion to the intensity of mass vaccination and vaccination in risk groups), and so on. Except for sensitive noninfected, recovered, or dead persons for all classes of variables, who have not switched to this moment to another class, the “age” of staying in this class is increased by one day, which brings them closer to the final disposal from the class. 

For more information, see Supplementary Data 1 and Supplementary Figure 1 available online at http://dx.doi.org/10.1155/2013/467078.

The model is web-available on the server of the SRC VB Vector and provided with a web interface (see Supplementary Figure 2), which allows a remote user to perform operations listed below:

- specifying a unique name for the session in order to avoid any conflicts with other users and to save their own data on the server for a certain time period;
- selecting an infectious agent from the default list; this option also allows the user to specify epidemic parameters for the agents absent from the list of modeled agents but of interest to user;
- accessing the review of published data on epidemiology, pathogenesis, prevention, and treatment of each disease from the list of modeled ones (only in Russian);
- editing parameters that specify the specific features of infection, the time of switching on AEA and their intensity, the population size and resource availability for a region (locality or a city) involved in simulation of an epidemic (outbreak), and the parameters necessary for computing the losses caused by the epidemic as well as supplies needed;
- computing the dynamics of epidemic; viewing/saving the results;
- calculating the losses caused by epidemic;
- calculating supplies needed;
- computing the dynamics of epidemic from a selected day (within the previously calculated period of its development). This regime can be helpful when certain considerable changes in the population take place during the development of epidemic; for example, if new infected cases come from (an)other region(s), thereby aggravating the situation, or, on the contrary, vaccines and drugs are conveyed from (an)other region(s) due to emergency situation, additional staff is engaged, or any other changes in the operational situation take place;
- computing four typical scenarios in development of an epidemic in a certain region in the case of a mass infection; and
- viewing a *User's Guide*, which describes how all these manipulations should be performed.

A certain default parameter set is defined for each of the modeled infections. This set corresponds to a scenario that is close to a “moderately optimistic” development of an epidemic for a “model” city.

The web-available manual contains detailed description of the model as well as descriptions of many options that can provide comfortable job for an epidemiologist, who has no modeling experience. 

## 3. Results and Discussion

Below, we illustrate the capacities of the proposed model by a case study of Ebola hemorrhagic fever (EHF). The data for adaptation of the model have been partially extracted from published reports [10–12] and partly from the plots reflecting manifestation of EHF symptoms during the 1995 Congo and 2000-2001 Uganda outbreaks [13, 14].

**Figure 1:**
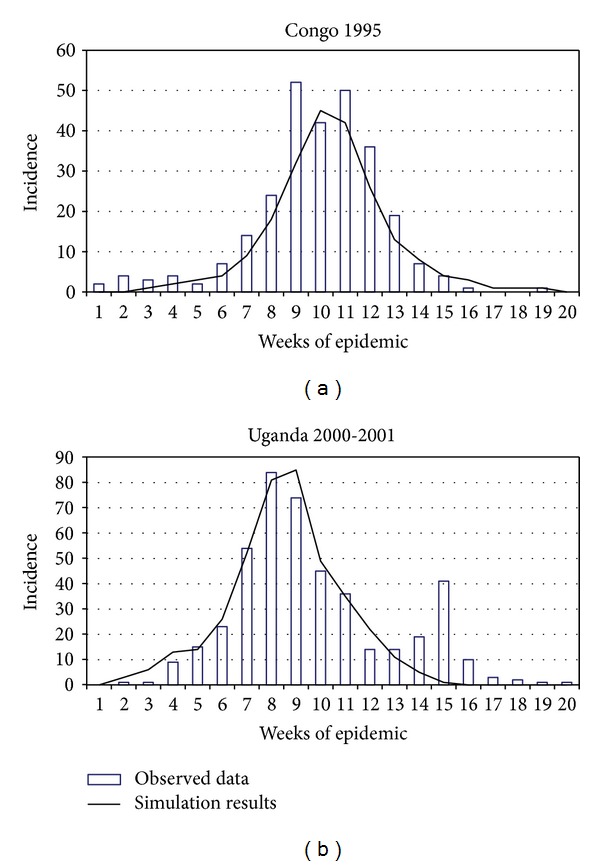
Observed data [14] and simulation results for the 1995 Congo epidemic and 2000 Uganda epidemic.

Despite the fact that the model is not intended for description of outbreaks with a small number of infected cases, to make the parameters more precise, we have had to use the data of real outbreaks where the initial number of cases was just several persons. In this procedure, we varied the outbreak characteristics, such as the initial number of infected case; the mean number of infected case from one patient without applying AEA, transmission speed *R*_0_; the time of AEA1 switch-on from the moment when the first infected cases were recorded; the mean number of contacts per one infected case; and the rate of infection of contacts. The main model parameters are listed in Supplementary Table 1. Note that these parameters are of an evaluative character and may be adjusted by experts.  Figure 1 shows the plots reflecting the appearance of EHF symptoms recorded in real outbreaks as well as the calculated values obtained as a result of model adaptation. Note also that characteristic of the EHF outbreak in Uganda is a certain periodicity [12], explainable only by a large number of primary cases that were infected almost simultaneously. Because durations of disease stages vary in too wide ranges and this is reflected in the model parameters (long period of transmission from one stage of illness to another), the model fails to reproduce this effect. For example, latent period durations for different patients vary from 2 to 20 days [11]. In addition, this periodicity itself can be an artifact reflecting the specificity of data collection and representation.

When simulating mass epidemics of Ebola fever in large European cities, let *R*_0_ retain the value of 2.5, since, on the one hand, the population density in cities is higher than it was in Congo and Uganda, and, on the other hand, the families (where the main part of infection transmission cases actually takes place) in cities are, as a rule, noticeably smaller. The collective immunity is specified at the level of 10%, which in our opinion may actually fit the natural background of decreased individual susceptibility to relatively small infective doses at random contacts between infected and healthy persons. The external infectious source is absent. At the initial time moment, the population contains 500 infected persons at the beginning of latent stage.

Resources of the model city and parameters controlling activation of countermeasures (AEA) are specified in the Table 1.

**Figure 2:**
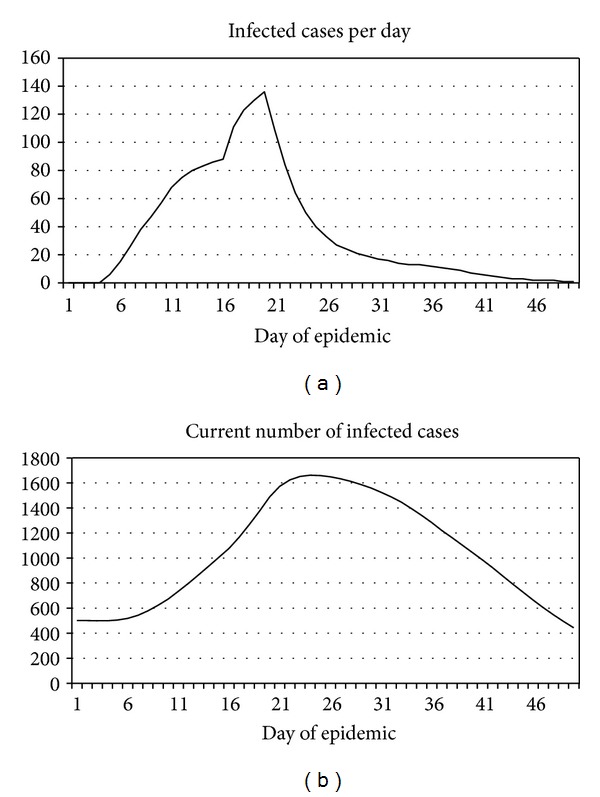
An example of computing the dynamics of Ebola fever outbreak with a “zero” parameter set.

An example of calculation of the EHF epidemic dynamics for this parameter set, for simplicity referred to as a “zero” set, is shown in Figure 2.

The computation protocol besides an epidemic dynamics outputs the following messages.

 
*Day  10*:* AEA1 are activated if infected cases are recorded *42 ≥ 30*. *
 
*Day  14*:* insufficient bed capacity for isolating patients 100. *
 
*Day  15*: *AEA2 are activated *15 ≥ 10 + 5*. *
 
*Day  18*:* insufficient bed capacity for isolating suspects 800. *
 
*Day  20*:* AEA3 are activated; resource limitations are canceled. *
 
*Day 20*:* quarantine is imposed 50%. *
  
*2313*: *total number of infected persons. *
 
*2047*: *total number of isolated persons. *
 
*1395*: *total number of observed contacts. *
 
*42020*: *total number of observed suspects. *
 
*13116*: *person days of observed contacts. *
 
*233398*: *person days of observed suspects. *
 
*19211*: *person days of isolated patients. *
 
*30*: *quarantine (days). *
 
*1504*: *total number of lethal cases. *

By day 50 of simulated epidemic, the outbreak does not end but, owing to the cancellation of the resource limitations is actually arrested, since almost all infected persons by that time have been isolated and there is no new infected cases. However, note that the number of infected cases per day decreases as early as by day 20, on the one hand, because the primary infected 500 persons by that time start to recover and, on the other hand, due to effectiveness of countermeasures.

**Figure 3:**
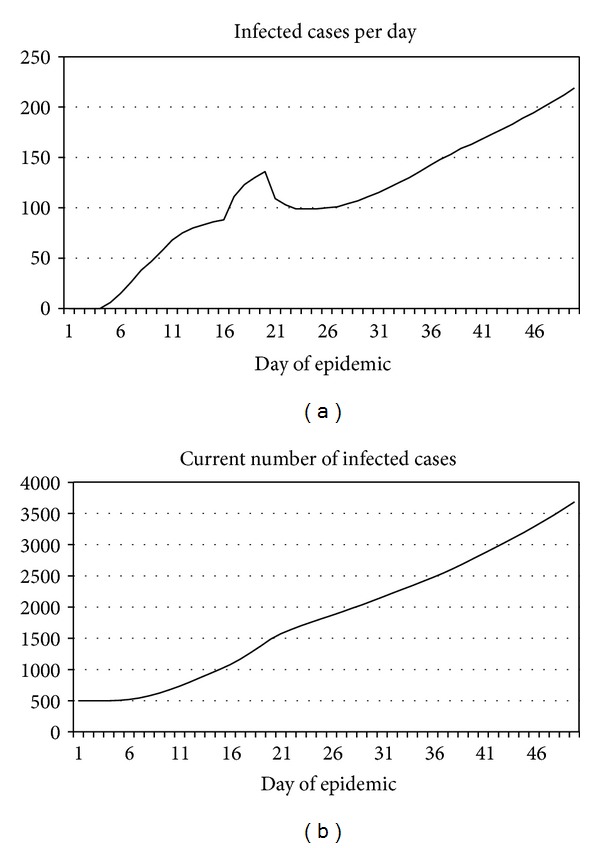
An example of computing the dynamics of Ebola fever outbreak under condition that the resource limitations are not cancelled at an AEA3 level (reference scenario).

The following “reference scenario” (Figure 3) differs from the previous one in that resource constraints are not canceled at an AEA3 level. The pattern has drastically changed. The outbreak is far from its end by day 50: the number of persons infected per day is over 200.

 The total number of infected persons during this time has reached over 6000 persons (and this number is increasing) versus a total of 2313 in the case of a “zero” scenario.

**Figure 4:**
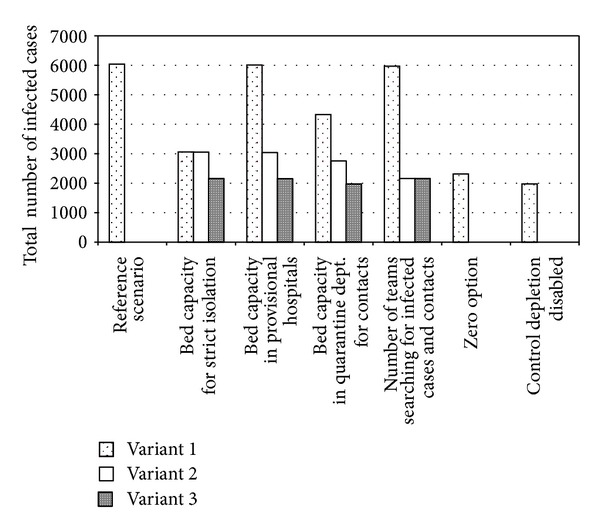
The effects of resource limitations on consequences of an epidemic (the number of infected cases): (i) Variant 1, the amount of each resource is increased tenfold as compared with a “zero” scenario; (ii) Variant 2, on the background of tenfold increased capacity for isolation of infected cases, the limitation on one of the remaining resources is canceled; (iii) Variant 3, on the background of no deficiency in isolation capacities and the teams searching for infected cases and contacts, the limitation on one of the remaining resources is canceled.

To test the effect of particular limitations on the outbreak dynamics, the amount of each resource was individually increased tenfold, that is, so that the limit for each resource would be unachievable.  Figure 4 shows the data on the total number of infected cases (Variant 1).

 The calculation results demonstrate that only an increase in the capacity for isolation of patients is capable of almost halving the number of infected persons over the period of 50 days (3058 versus 6038). An increase in the capacity for isolation of contacts also gives a noticeable although not so considerable effect (4327 infected cases). The influences of all the remaining factors are significantly smaller.

On the background of a tenfold increase in the capacities for isolation of patients (100 ⇒ 1000), the limitations for only one resource of those remaining that were in deficit were canceled. In this case, the best result—2160 infected cases —was obtained when there was no deficiency in the teams searching for infected cases and contacts, that is, when there was the possibility to isolate and treat them not only to a full degree, but also in good time (Figure 4, Variant 2).

Canceling the condition for deficit in the capacities for isolation of patients and in medical teams, we again individually increased the amount of each resource. In this case, the maximal effect is observed when increasing the capacities for isolation of contacts. The result is even better than in the case of a “zero” variant and almost from the same as the calculations with no resource deficiencies at all: the total numbers of infected persons are 1977 and 1971, respectively (Figure 4, Variant 3).

**Figure 5:**
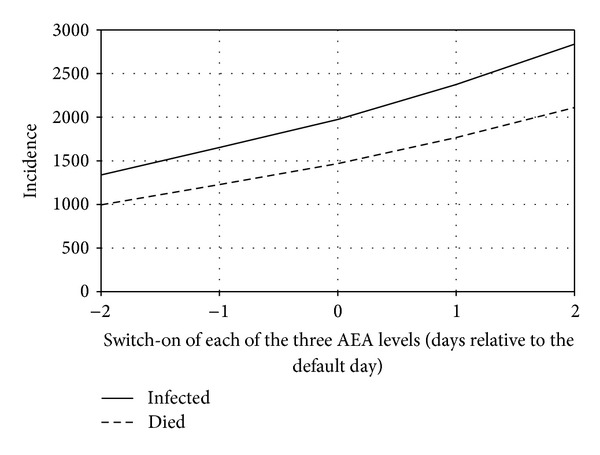
Dependence of the number of infected and lethal cases on day 50 of epidemic on the day when AEA were switched on in the absence of any resource limitations.

**Figure 6:**
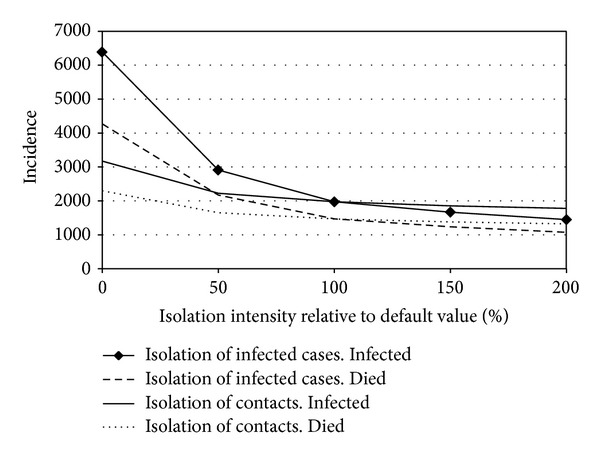
Dependence of the number of infected and lethal cases on day 50 of epidemic on the intensity of isolation of infected cases and contacts in the absence of any resource limitations.

In addition to the considered factors, the moment of activation on AEA and their intensity are also important for the dynamics of an epidemic (Figures 5 and 6).

We purposefully do not illustrate the effect of drug resource, since it influences only the level of mortality and has no effect on the dynamics of the number of infected persons. Factors, such as observation/isolation of the persons displaying nonspecific disease symptoms being uninfected, are according to our computations rather resource intensive and, thereby, have an indirect effect on the epidemic dynamics.

All the shown computations have been performed in a remote mode on the server of SRC VB Vector. It is web-available, so that any user may repeat them or conduct their own computations of interest.

## 4. Conclusions

A universal model describing dynamics of mass epidemics (outbreaks) of the socially significant diseases as well as infections caused by special pathogens is being developed at the State Research Center of Virology and Biotechnology Vector. The range of major interventions is provided in the model, including preventive and emergency mass vaccination, vaccination of risk groups as well as search for and isolation or observation of infected cases, contacts, and suspects, and quarantine. For computations, it is possible to specify the parameters that determine the resources specific of a city (or a region) deficiency which can influence the intensity of countermeasures. The results of calculation, based on, for example, *Person days of observed contacts*, *Person days of observed suspects,* and *Person days of isolated patients* and current values of several variables make it possible to estimate losses from an epidemic and the material and human resources required for eradication of an epidemic (supplies).

## Supplementary Material

The supplementary materials contain some description of the epidemic model in continuous form, main model parameters characteristic for Ebola fever, a scheme of transitions between the main variables of the model, taking into account some countermeasures, and the screenshot of the website main page.

## Figures and Tables

**Table 1:** Some parameters used for calculations of an EHF outbreak dynamics.

Resources of the model city	
Population	500000
Number of medics/paramedics involved in AEA	1000
Number of teams searching for and isolating infected cases and contacts	30
Number of patients/contacts detected by one team per day	20
Reserve of drugs (for one treatment course)	1000
Bed capacity for strict isolation	100
Bed capacity in provisional hospitals	800
Bed capacity in quarantine departments for contacts	300

Parameters controlling activation of countermeasures (AEA)	

AEA1 is activated	
(i) At a calculated critical time moment (days of epidemic) or	20
(ii) When the number of infected persons in the final stage reaches a critical value	30
Delay in activation of AEA2 relative to AEA1 (days)	5
Delay in activation of AEA3 relative to AEA2 (days)	5
Activation of quarantine after AEA2 (days)	5
Intensity of quarantine (%)	50
Number of days without detection of any infected persons to cancel quarantine	15
Control of resource depletion	On
Resource limitations in AEA3	Canceled
