# Supplementary material for: A Universal Model for Predicting Dynamics of the Epidemics Caused by Special Pathogens
Source: Biomed Res Int. 2013 Jul 24;2013:467078. doi: 10.1155/2013/467078 (PMC3741903; doi:10.1155/2013/467078)
Supplement: Supplementary file 1 — The supplementary materials contain some description of the epidemic model in continuous form, main model parameters characteristic for Ebola fever, a scheme of transitions between the main variables of the model, taking into account some countermeasures, and the screenshot of the website main page. [file 467078.f1.pdf]

Supplementary Material

Supplementary Data 1: Description of the model in continuous form.

Let it be:

t - a time from the start point of calculations;

ν - immunity level (generally continuous, but really used 0 or 1 values in this version of the model);

τ - the residence time at some stage of the disease;

N(t) - population;

$S(\nu,t)$ - non-infected sensitive individuals with the level of immunity ν;

$S_{con}(\nu,t)$ - non-infected sensitive individuals, contacted with infected ones, not detected;

$S_c(\nu,t)$ - non-infected sensitive individuals, contacted with infected ones, detected and observed or isolated;

$S_{sus}(\nu,t)$ - non-infected sensitive individuals, displaying primary nonspecific disease symptoms detected and observed or isolated (suspects);

$S_v(\zeta,t)$ - non-infected sensitive persons during establishment of immunity after vaccination $\nu: 0 \rightarrow 1$, with the residence time at this stage τ;

$L(\nu,\tau,t)$ - persons in a latent stage;

$L_c(\nu,\tau,t)$ - persons in a latent stage, detected and observed or isolated as contacts;

$L_{suc}(\nu,\tau,t)$ - suspected persons in a latent stage, detected and observed or isolated;

$L_v(\zeta,\tau,t)$ - persons in a latent stage during establishment of immunity after vaccination

$P(\nu,\tau,t)$ - persons in the second stage of disease (usually, prodromal stage), not detected;

$P_i(\nu,\tau,t)$ - persons in the second stage of disease (usually, prodromal stage), detected and observed or isolated;

$I_h(\nu,\tau,t)$ - persons in the final stage of disease, not detected, displaying a severe form of disease;

$I_l(\nu,\tau,t)$ - persons in the final stage of disease, not detected, displaying a mild form of disease;

$I_{ih}(\nu,\tau,t)$ - persons in the final stage of disease, which are observed or isolated, displaying a severe form of disease;

$I_{il}(\nu,\tau,t)$ - persons in the final stage of disease, which are observed or isolated, displaying a mild form of disease;

V(t) - "infection stress" (intensity of infection) because of a potential external infection source and/or infectivity of not isolated infected cases at the second and/or third stages of disease.

Then

$$dV(t)/dt = \alpha(t) - \kappa(t) \times V(t). \quad (1)$$

Here

$$\alpha(t) = \Sigma(\int(\int\gamma_q(\nu,\tau)\times I_q(\nu,\tau,t)\, d\tau + \int\lambda_q(\nu,\tau)\times P_q(\nu,\tau,t)\times d\tau)\times d\nu) + \varphi(t) - \quad (2)$$

recharge rates of infectious pool from all sources: $\lambda_q(\nu,\tau)$ and $\gamma_q(\nu,\tau)$ - infectivity of ill persons of ***q***-classes (patients in prodromal and hemorrhagic stages, which are not isolated); $\varphi(t)$ is a possible completion of the pool from some external sources.

In all equations **Σ** means the summation over all relevant ***q***-classes.

Processes of infection can be in general described as

$$dS_q(\nu,t)/dt = -l(t)\times\beta_q(\nu)\times V(t)\times S_q(\nu,t)$$

$$dL_q(\nu,0,t)/dt = l(t)\times\beta_q(\nu)\times V(t)\times S_q(\nu,t)$$

$$dS_{con}(\nu,t)/dt = c\times l(t)\times\beta_q(\nu)\times V(t)\times S_q(\nu,t) \quad (3)$$

Here $\beta_q(\nu)$ - a sensitivity to infection of q-class persons (including contacts, suspects, and so on), $l(t)$ is a reduction quarantine factor, c - an average number of contact persons for each the infected.

Convalescence is defined by simple expressions of the form

$$dR(t)/dt = \Sigma(\int(\int\rho_q(\nu,\tau)\times I_q(\nu,\tau,t)\times d\tau)\times d\nu)$$

$$dI_q(\nu,\tau,t)/dt = -\rho_q(\nu,\tau)\times I_q(\nu,\tau,t) \quad (4)$$

Similar relations are given for mortality

$$dD(t)/dt = \Sigma(\int(\int\delta_q(\nu,\tau)\times I_q(\nu,\tau,t)\times d\tau)\times d\nu)+\Sigma(\int(\int\delta_q(\nu,\tau)\times P_q(\nu,\tau,t)\times d\tau)\times d\nu)$$

$$dP_q(\nu,\tau,t)/dt = -\delta_q(\nu,\tau)\times P_q(\nu,\tau,t)$$

$$dI_q(\nu,\tau,t)/dt = -\delta_q(\nu,\tau)\times I_q(\nu,\tau,t) \quad (5)$$

Let $U_q(\nu,\tau,t)$ is a number of infected persons, which come τ days to the stage of disease, and $\sigma_q(\nu,\tau)$ is an integral function of "departure" from this stage due to the transition to the next stage, convalescence, death, hospitalization, etc. Then development of a stage of the disease can be written by the following equation:

$$\partial U_q(\nu,\tau,t)/\partial\tau + \partial U_q(\nu,\tau,t)/\partial t = -\sigma_q(\nu,\tau)\times U_q(\nu,\tau,t) \quad (6)$$

where $U_q(\nu,\tau,t0) = U0_q(\nu,\tau)$ is the initial condition.

The transition from the previous (***U***) to the next stage of the disease (***U '***) can be written the following equation:

$$dU'_q(\nu,0,t)/dt = \int\pi_q(\nu,\tau)\times U_q(\nu,\tau,t)\times d\tau$$

$$dU_q(\nu,\tau,t)/dt = -\pi_q(\nu,\tau)\times U_q(\nu,\tau,t) \quad (7)$$

Here $\pi_q(\nu,\tau)$ is transition function from the previous to the next stage for ***q***-class individuals.

Detection of ill, contact or suspected persons with following isolation or observation can be written as

$$dU_{iq}(\nu,\tau,t)/dt = \chi_q(\nu,\tau,t)\times U_q(\nu,\tau,t)$$

$$dU_q(\nu,\tau,t)/dt = -\chi_q(\nu,\tau,t)\times U_q(\nu,\tau,t) \quad (8)$$

Here $U_q(\nu,\tau,t)$ – ***q***-class of persons who have not been detected, $U_{qi}(\nu,\tau,t)$ – the relevant class of persons identified.

Suppose that a vaccination program q_vac (t) is given, expressed in the daily number of vaccinated persons in the sensitive group with $\nu < \nu_{max}$. Then

$$dS_q(\nu<\nu_{max},t)/dt = q\_vac(t)\times S_q(\nu<\nu_{max},t)/\Sigma\int S_q(\nu<\nu_{max},t)\times d\nu$$

$$dSv_q(0,t)/dt=\int S_q(\nu<\nu_{max},t)\times d\nu$$

$$\partial Sv_q(\zeta, t)/\partial\zeta + \partial Sv_q(\zeta, t)/\partial t = -\delta\_vac_q(\zeta)\times Sv_q(\zeta\ t)$$

$$dS_q(\nu,t)/dt = \int\delta\_vac_q(\zeta)\times Sv_q(\zeta, t)d\zeta \quad (9)$$

Here $\delta\_vac_q(\zeta)$ is a function of establishment of immunity for ***q***-class in dependence of ζ that is a time from the moment of vaccination.

SUPPLEMENTARY TABLE 1: Main parameters characteristic for Ebola fever

(Note that these parameters are of an evaluative character and may be adjusted by experts.)

Parameters	Value
Mean number of persons infected by one patient, R_0	2.5
Maximum duration of latent stage (days)	14
Maximum duration of initial stage (days)	7
Maximum duration of haemorrhagic stage (days)	16
Days while disease passes from one stage to the other:<br>latent stage => initial stage<br>initial stage => haemorrhagic stage<br>recovery	 2-14<br>5-7<br>12-16
Mean number of contacts per one infected person	3
Rate of infectious activity in haemorrhagic stage, (%)	70
Ratio of infectious activity of immune to nonimmune, (%)	50
Ratio of infectious activity of cases with mild to severe forms, (%)	20
Rate of severe forms among nonimmune patients, (%)	90
Rate of severe forms among immune patients, (%)	10
Ratio of sensitivity to infection of immune persons relative to nonimmune, (%)	20
Rate of infected contacts per day, (%)	20
Mortality rate of nonimmune patients in initial stage, (%)	10
Mortality rate of immune patients in initial stage, (%)	5
Mortality rate of nonimmune patients with severe form in haemorrhagic stage, (%)	90
Mortality rate of immune patients with severe form in haemorrhagic stage, (%)	10
Mortality rate of immune patients with mild form in haemorrhagic stage, (%)	0.3
Decrease in mortality rate of treated cases relative to untreated, (%)	80
Observation duration for contacts (days)	21

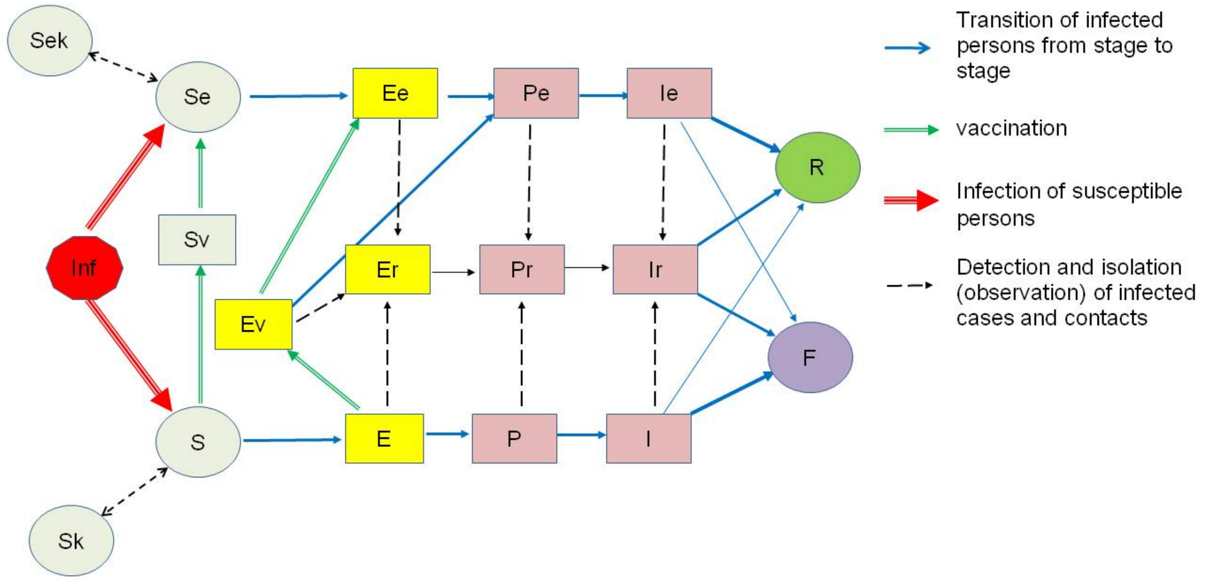

S, Se - Uninfected persons susceptible to infection: nonimmune and immune;
Sk, Sek - Detected and observed/isolated contacts : nonimmune and immune;
Sv - Uninfected persons during establishment of immunity after vaccination;
E, Ee, Er, Ev - Infected persons in a latent stage: nonimmune , immune, observed/isolated and vaccinees;
P, Pe, Pr - Infected persons in the second stage of disease (usually, prodromal stage), nonimmune, immune and observed/isolated;
I, Ie, Ir - Infected persons in the final stage of disease : nonimmune , immune and observed/isolated (displaying a mild or a severe form of disease);
R - The persons that have recovered ;
F - Those who died of disease;
Inf - "infection stress" (intensity of infection).

SUPPLEMENTARY FIGURE 1: Transitions between the main variables of the model, taking into account some countermeasures.

In the stages marked by rectangles, the state of the infected people and transition probabilities to the next stage depends on the time stay in this stage ("age"). The states of the variables marked by circles are independent of time.

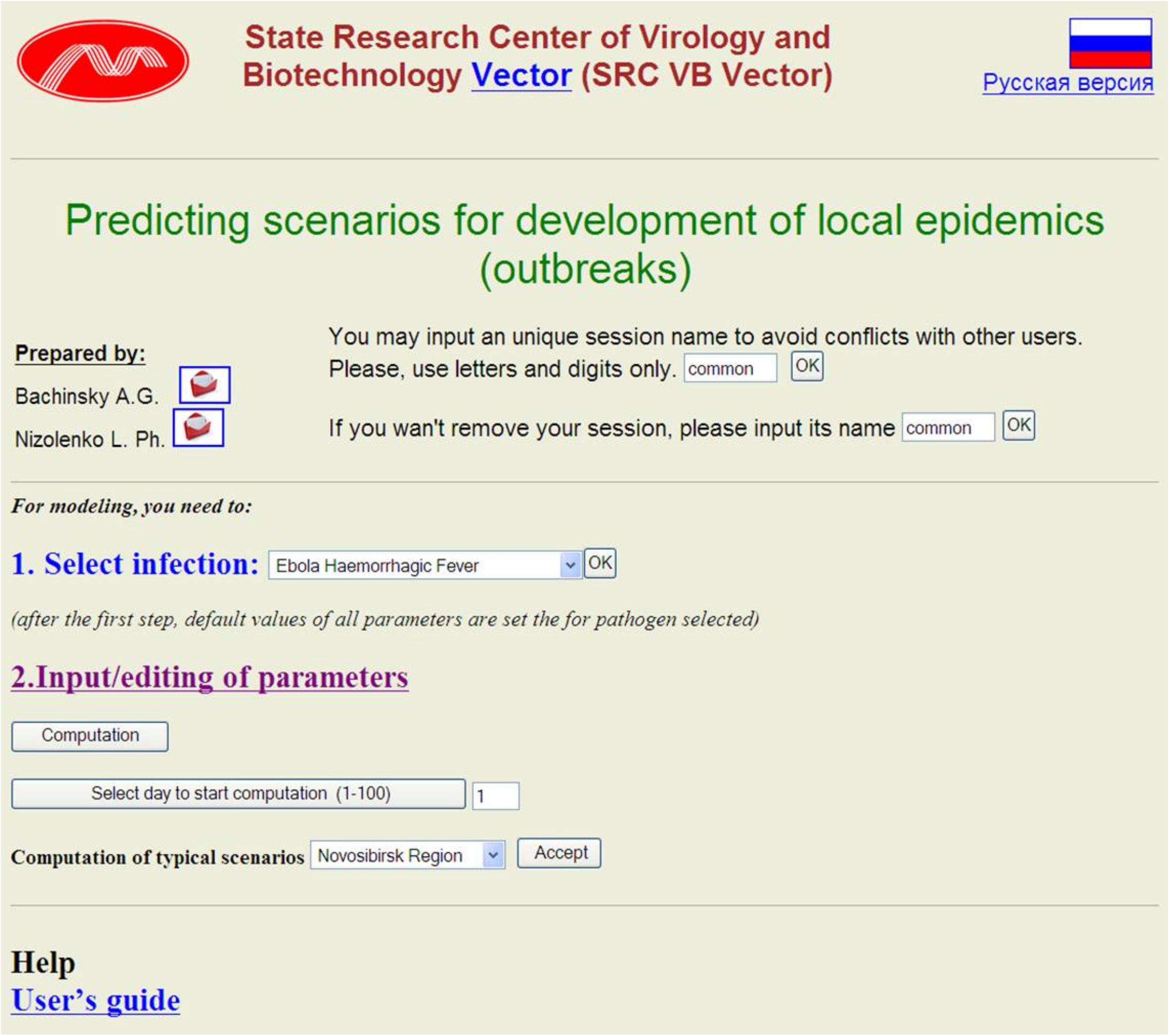

SUPPLEMENTARY FIGURE 2: The screenshot of the website main page
